# Swine Host Protein Coiled-Coil Domain-Containing 115 (CCDC115) Interacts with Classical Swine Fever Virus Structural Glycoprotein E2 during Virus Replication

**DOI:** 10.3390/v12040388

**Published:** 2020-03-31

**Authors:** Elizabeth A. Vuono, Elizabeth Ramirez-Medina, Keith Berggren, Ayushi Rai, Sarah Pruitt, Ediane Silva, Lauro Velazquez-Salinas, Douglas P. Gladue, Manuel V. Borca

**Affiliations:** 1 Plum Island Animal Disease Center, ARS, USDA, Greenport, NY 11944, USA; Elizabeth.Vuono@usda.gov (E.A.V.); keith.a.berggren@gmail.com (K.B.); ayushi.rai@usda.gov (A.R.); sarah.pruitt@usda.gov (S.P.); ediane.silva@usda.gov (E.S.); Lauro.velazquez@usda.gov (L.V.-S.); 2 Department of Pathobiology and Population Medicine, Mississippi State University, P.O. Box 6100, Starkville, MS 39762, USA; elizabeth.ramirez@usda.gov; 3 Department of Pathobiology and Veterinary Science, University of Connecticut, Storrs, CT 06269, USA; 4 Oak Ridge Institute for Science and Education (ORISE), Oak Ridge, TN 37830, USA; 5 Department of Anatomy and Physiology, Kansas State University, Manhattan, KS 66506, USA

**Keywords:** swine fever viruses, CSFV, classical swine fever

## Abstract

Interactions between the major structural glycoprotein E2 of classical swine fever virus (CSFV) with host proteins have been identified as important factors affecting virus replication and virulence. Previously, using the yeast two-hybrid system, we identified swine host proteins specifically interacting with CSFV E2. In this report, we use a proximity ligation assay to demonstrate that swine host protein CCDC115 interacts with E2 in CSFV-infected swine cells. Using a randomly mutated E2 library in the context of a yeast two-hybrid methodology, specific amino acid mutations in the CSFV E2 protein responsible for disrupting the interaction with CCDC115 were identified. A recombinant CSFV mutant (E2ΔCCDC115v) harboring amino acid changes disrupting the E2 protein interaction with CCDC115 was produced and used as a tool to assess the role of the E2–CCDC115 interaction in viral replication and virulence in swine. CSFV E2ΔCCDC115v showed a slightly decreased ability to replicate in the SK6 swine cell line and a greater replication defect in primary swine macrophage cultures. A decreased E2–CCDC115 interaction detected by PLA is observed in cells infected with E2ΔCCDC115v. Importantly, animals intranasally infected with 10^5^ TCID_50_ of E2ΔCCDC115v experienced a significantly longer survival period when compared with those infected with the parental Brescia strain. This result would indicate that the ability of CSFV E2 to bind host CCDC115 protein during infection plays an important role in virus replication in swine macrophages and in virus virulence during the infection in domestic swine.

## 1. Introduction

Classical swine fever virus (CSFV) is the causative agent of a highly contagious disease of swine with important economic consequences. CSFV is a small enveloped virus harboring a positive single-stranded RNA genome of approximately 12.5 kb. CSFV encodes a single open reading frame polyprotein composed of 3898 amino acids, which, after post-translational cleavage, yields 11–12 final products (NH2-Npro-C-Erns-E1-E2-p7-NS2-NS3-NS4A-NS4B-NS5A-NS5B-COOH) [1].

Classical swine fever (CSF) is endemic in Central and South America, Asia and parts of Africa and, just recently, after being free of the disease for twenty-six years, CSF was detected in wild boar and domestic pigs in Japan [2]. The C-strain vaccine, an attenuated CSFV developed by serial passage in rabbits, has been used effectively against CSF disease. Recent reports have suggested that the emergence of field strains in China and Cuba may represent escape viral variants that the C-strain vaccine does not provide protection against [3]. 

CSFV particles are formed by four structural proteins—the Core protein that surrounds the virus genome, and three envelope glycoproteins, E^rns^, E1 and E2. Understanding the molecular function of these proteins, along with the identification of specific amino acid residues involved, in processes such as virus replication and virulence has been a focus in recent years [4,5,6,7,8,9,10]. Therefore, the identification of host cell proteins specifically interacting with these virus proteins during infection is a relatively new field of CSFV research. CSFV Core protein has been shown to interact with IQ motif-containing GTPase activating protein 1 (IQGAP1), ubiquitin-conjugating enzyme 9 (UBC9), small ubiquitin-related modifier 1 (SUMO1), and hemoglobin subunit beta (HB) [11,12,13,14]. E^rns^ has been demonstrated to interact with the Laminin receptor [15]. In addition, E2 has been shown to interact with several host proteins including cellular actin [16], thioredoxin [17], annexin 2 [18], mitogen-activated protein kinase 2 [19], protein phosphatase 1 catalytic subunit beta [20] and dynactin 6 [21]. These host–virus protein–protein interactions have been shown to play a role in regulating the virus replication cycle and, in some cases, these protein interactions are involved in virus virulence [11,12,13,21].

Using a yeast two-hybrid approach, we identified several swine host proteins specifically interacting with CSFV E2 [22]. Here, we expand our preliminary report by analyzing the protein–protein interaction between one of those host proteins, CCDC115, and CSFV E2. CCDC115 is a coiled-coil domain-containing protein, with a largely unknown cellular function. CCDC115 localizes in the endoplasmic reticulum (ER)-Golgi intermediate compartment and coat protein complex I vesicles in some human cells. A missense mutation in the CCDC115 protein has been implicated in a disorder of Golgi homeostasis in humans, with abnormalities in the process of protein glycosylation [23]. Here, using proximity ligation assay (PLA), we demonstrated that the interaction between these two proteins occurs in CSFV-infected cells. The E2 amino acid residues critical for disrupting that interaction were mapped by yeast two-hybrid and, based on that information, a recombinant CSFV mutant (E2ΔCCDC115v) was developed by reverse genetics harboring residue mutations disrupting the E2–CCDC115 interaction. CSFV E2ΔCCDC115v replicates well in the swine cell line SK6 but less efficiently in primary swine macrophages. Interestingly, animals intranasally infected with 10^5^ TCID_50_ of E2ΔCCDC115v showed a significantly longer survival period when compared with those infected with the parental Brescia strain. This result would indicate that the ability of CSFV E2 to bind host CCDC115 protein during infection plays an important role in virus replication in swine macrophages and in virus virulence during the infection in domestic swine.

## 2. Materials and Methods 

### 2.1. Viruses and Cells 

Swine kidney cells (SK6, Center for Veterinary Diagnostics, Ames, Iowa), free of BVDV (bovine viral diarrhea virus), were cultured in Dulbecco’s Minimal Essential Media (DMEM) (Gibco, Grand Island, NY, USA) with 10% fetal calf serum (FCS) (Atlas Biologicals, Fort Collins, CO, USA). The CSFV Brescia strain was propagated in SK6 cells and used for the construction of an infectious cDNA clone (IC) [4]. Growth kinetics was assessed on both SK6 cells and primary swine macrophage cell cultures prepared as described [4]. Titration of CSFV from clinical samples was performed using SK6 cells in 96-well plates (Costar, Cambridge, MA, USA). Viral infectivity was assessed after 4 days in culture by an immunoperoxidase assay using the CSFV monoclonal antibody WH303 [24] and the Vectastain ABC kit (Vector Laboratories, Burlingame, CA, USA). Titers were calculated as previously described [25] and expressed as tissue culture infectious doses per milliliter (TCID_50_/_mL_). As performed, test sensitivity was ≥ 1.8 TCID_50_/_mL_. Plaque assays were performed using SK6 cells in 6-well plates (Costar). SK6 monolayers were infected, overlaid with 0.5% agarose and incubated at 37 °C for 3 days. Plates were fixed with 50% (*v*/*v*) ethanol/acetone and stained by immunohistochemistry with monoclonal antibody WH303 [24].

### 2.2. Proximity Ligation Assay

The proximity ligation assay (PLA) was performed exactly as described elsewhere [21]. Briefly, the assay was run in triplicate following guidelines from the Duolink-PLA kit (Sigma-Aldrich). SK6 cells were plated onto 12 mm round coverslips (Thomas Scientific, Swedesboro, NJ, USA) in a 24-well plate (Corning, Corning, NY, USA) at a density of 25,000 cells/well. Next day, cells were infected (MOI = 10) and, 24 h later, cells were fixed with 4% formaldehyde *w*/*v* in PBS at room temperature for 20 min, followed by permeabilization buffer (0.3% Triton-X-100 in PBS) for 10 min. Fixed cells were then blocked with Duolink blocking buffer for 30 min at 37 °C, followed by incubation with primary antibodies, anti-E2 WH303 [24] and anti-CCDC115 (Invitrogen Cat # PA5-56987), at 4 °C for 1 h. Cells were then washed twice with Duolink (Sigma-Aldrich) wash buffer A and incubated with the PLUS and MINUS PLA probes for 1 h at 37 °C, followed by 2 washes with Duolink wash buffer A, and a 30 min incubation at 37 °C with Duolink ligase in ligation buffer. Fixed cells were then washed twice with Duolink wash buffer A, followed by incubation with Duolink polymerase in amplification buffer at 37 °C for 100 min. The fixed cells were then washed twice with Duolink wash buffer B and mounted with Duolink PLA Mounting Medium with DAPI.

### 2.3. Yeast Two-Hybrid Screening for Disruption of CCDC115 Binding E2

Plasmids E2-BD and CCDC115-AD were previously identified or constructed [22]. E2-BD was randomly mutated using a mutagenic PCR approach to give an average of 5 nucleotide substitutions across the E2 open reading frame (this random mutant library was constructed by Epoch Bioscience (Bothell, WA, USA). The random mutant library was then co-transformed into yeast strain AH109, along with CCDC115-AD, with a transformation rate of at least 1 × 10^6^ individual colonies representing full coverage of the E2 mutagenic library. Screening for disruption of E2-binding domains for CCDC115-AD was performed as previously described [21]. Sequencing of disruption of binding mutants in E2 often revealed stop codons or out-of-frame mutations, thus explaining why the loss of the E2–CCDC115 interaction occurred—these plasmids were discarded. When individual amino acids were mutated, the E2 mutant plasmids were tested by co-transformation with CCDC115 and hypoxanthine phosphoribosyl transferase 1 (HPRT1-AD), a previously identified [22] positive E2 protein interactor, or PGADT7 (negative control) and selected on SD-TL plates. Then individual colonies were grown overnight in SD-TL liquid media at 30 °C and spot plated on both SD-TL and SD-ALTH to assess the ability of individual E2 mutants to bind both positive and negative controls (HPRT1-AD and PGADT7, respectively) and to confirm loss of binding to CCDC115. This second selection was performed to discard any mutant E2 proteins that lost the ability to bind CCDC115 because of a gross structural change in E2, and not loss of a critical residue for binding CCDC115.

### 2.4. Construction of CSFV E2ΔCCDC115v Mutant

A full-length IC of the virulent CSFV Brescia strain (pBIC) [4] was used as a template in which E2 amino acid substitutions disrupting the E2–CCDC115 interaction, as mapped by reverse yeast two-hybrid methodology, were included. Residue substitutions, K112M, N260I, and M275S, were introduced into the native E2 sequence in the designed pBICΔCCDC115 construct. The pBICΔCCDC115 plasmid was obtained by DNA synthesis (Epoch Life Sciences, Sugar Land, TX, USA). 

The CSFV pBICΔCCDC115 full-length genomic clone was linearized with SrfI and in vitro transcribed using the T7 MEGAscript system (Ambion, Austin, TX, USA). RNA was precipitated with LiCl and transfected into SK6 cells by electroporation at 500 volts, 720 ohms, and 100 watts, with a BTX 630 electroporator (BTX, San Diego, CA, USA). Cells were seeded in 12-well plates and incubated for 4 days at 37 °C and 5% CO^2^. Virus was detected by immunoperoxidase staining as described above, and stocks of rescued viruses were stored at ≤ −70 °C. Full-length genome of in vitro rescued CSFV E2ΔCCDC115 virus (E2ΔCCDC115v) was completely sequenced by NGS.

### 2.5. Ethics Statement

Animal experiments were performed under biosafety level 3AG conditions in the animal facilities at Plum Island Animal Disease Center (PIADC). All experimental procedures were carried out in compliance with the Animal Welfare Act (AWA), the 2011 Guide for Care and Use of Laboratory Animals, the 2002 PHS Policy for the Humane Care and Use of Laboratory Animals, and U.S. Government Principles for Utilization and Care of Vertebrate Animals Used in Testing, Research and Training (IRAC 1985), as well as specific animal protocols reviewed and approved by the PIADC Institutional Animal Care and Use Committee of the US Departments of Agriculture and Homeland Security (protocol number 171.05-18-R Classical swine fever virus (CSFV): evaluation of virulence of wild-type and genetically modified viruses; approved on 02-10-18). 

### 2.6. Animal Infections

E2ΔCCDC115v mutant was evaluated for its virulence phenotype in swine relative to the virulent Brescia strain. Swine used in all animal studies were 10–12-week-old, forty-pound commercial-breed pigs. Five animals were inoculated intranasally with 10^5^ TCID_50_ of either E2ΔCCDC115v or wild-type parental virus (BICv). Clinical signs (anorexia, depression, purple skin discoloration, staggering gait, diarrhea and cough) and changes in body temperature were recorded daily throughout the 21 day experiment. Total and differential white blood cell, lymphocyte and platelet counts were obtained using a Hemavet HV950FS (Drew Scientific Inc, Miami Lakes, FL, USA).

## 3. Results

### 3.1. CSFV E2 and Swine Host Protein CCDC115 Interact in CSFV-Infected Cells

We have already reported the interaction of swine host CCDC115 with CSFV E2 discovered using a yeast two-hybrid approach using a custom-made library [22]. In addition, the E2–CCDC115 interaction was shown to involve noncontinuous binding areas in E2. Previous experiments have shown that a library of E2 mutants harboring substitutions of native residues by stretches of alanine residues failed to identify a linear specific area in E2 mediating the binding with CCDC115 protein [22]. To confirm that the interaction between E2 and CCDC115 takes place during CSFV infection, we used a proximity ligation assay (PLA) [26].

**Figure 1:**
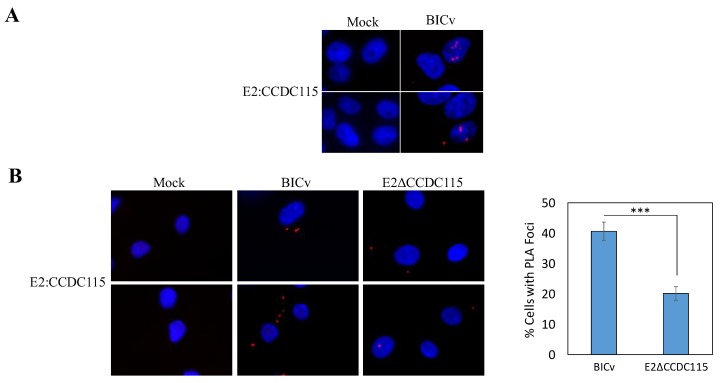
Interaction between CSFV E2 and CCDC115 in CSFV-infected cells demonstrated by proximity ligation assay (PLA). Images were visualized at 40X magnification. SK6 cells (**A**) or primary swine macrophages (**B**) that were either mock infected (Mock) or infected for 24 h with CSFV parental BICv or recombinant E2ΔCCDC115v (MOI = 10) as described in Materials and Methods. Panel B (on the right), quantification of the decrease in the E2-CCDC115 interaction in swine macrophages infected with either BICv or E2ΔCCDC115v, error bars represent the average and standard deviation from from the counting of three independent counts of 100 cells. *** *p*-value < 0.0008.

PLA, which allows the identification of transient protein–protein interactions, was performed using the E2-specific monoclonal antibody WH303 [24] and a CCDC115-specific commercially available monoclonal antibody. The CSFV Brescia strain (derived from an infectious clone encoding for Brescia, a highly pathogenic strain of CSFV) was used to infect (MOI = 10) either SK6 cell monolayers or primary swine macrophages cultures. Samples were harvested at 24 h post-infection (hpi) and processed as described in Materials and Methods. Results of sPLA confirmed that E2 and CCDC115 interact in SK6 and macrophage cell cultures infected with CSFV. This interaction appears as a distinct punctate location (Figure 1). These results indicated that in CSFV-infected cells E2 specifically interacts with CCDC115 in both SK6 cells and swine macrophages, confirming the previous yeast two-hybrid finding.

### 3.2. Identification of CSFV E2 Residues Critical for CCDC115 Interaction

To develop a tool to study the effect of disrupting the E2–CCDC115 interaction on CSFV functions, it is important to design a recombinant CSFV with an altered ability of E2 to specifically recognize CCDC115. A precondition of developing such a recombinant virus is identification of the E2 amino acid residues responsible for that interaction. These mutant viruses, containing residue substitutions disrupting interactions between a virus protein and its host cell partner, have been used to analyze the potential role of a virus protein and host protein interaction in several virus functions. We have successfully used this approach to discover specific residues within foot and mouth disease virus (FMDV) and CSFV proteins interacting with host protein ligands [13,27,28,29,30]. 

**Figure 2:**
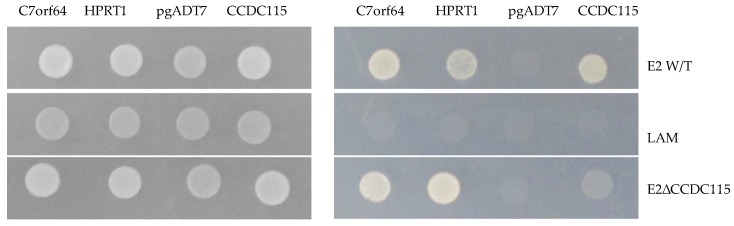
Yeast two-hybrid reactivity of E2 and E2ΔCCDC115. The indicated plasmids were transformed into the yeast strain AH109 and spotted on the left on -Trp/-Leu plates for plasmid selection and on the right on selective media -Trp/-Leu/-Ade/-His. C7orf64, HPRT1 and CDC115 are expressed with the activation domain for the yeast two-hybrid in the pgADT7 vector. pgADT7 is used as a negative control. E2 W/T, Lam and E2∆CDC115 are expressed with the binding domain for the yeast two-hybrid. Lam is used as a negative control.

As described earlier, we have already reported that the E2–CCDC115 interaction appears to be conformation dependent [22]. Therefore, to discover E2 amino acids mediating the interaction with CCDC115, we used an alternative approach using the yeast two-hybrid system already utilized in our laboratory [21], which allows the identification of E2 residues involved in the interaction with host cell ligands, even if they are not adjacently located. The approach assesses the capacity of swine CCDC115 to interact with a library of randomly mutated forms of E2 containing an average of five amino acid residue substitutions. Consequently, 1 × 10^6^ mutated forms of E2 were assessed in the yeast two-hybrid library screening already described in Materials and Methods. As a control, E2 mutants lacking reactivity with CCDC115 were also tested in their ability to bind protein HPRT1 and protein C7orf64—both are other host proteins that also specifically bind E2—to exclude the possibility that E2 residue replacements may cause important conformational modifications in E2 altering its overall structure, leading to a nonspecific loss of protein recognition. One E2 mutant was identified, harboring substitutions at positions K112M, N260I, and M275S, and it was used to further analyze the potential role for the E2–CCDC115 interaction in CSFV replication and virulence (Figure 2).

### 3.3. Development of CSFV E2ΔCCDC115v Mutant 

In order to understand the role of the E2–CCDC115 interaction in virus replication in vitro and in vivo, a recombinant mutant virus was developed by reverse genetics using an infectious clone encoding for the virulent strain Brescia. Based on the information obtained by disrupting the E2–CCDC115 interaction in the yeast two-hybrid system, a recombinant CSFV was designed (E2ΔCCDC115v) containing substitutions in the amino acid residues of E2 identified as necessary for the E2–CCDC115 interaction: K112M, N260I, and M275S. The infectious clone harboring the desired E2 mutations (pE2ΔCCDC115) was used to produce the infectious RNA by in vitro transcription followed by transfection of SK6 cells. CSFV E2ΔCCDC115v was rescued (along with the parental Brescia virus, BICv) from transfected cells by 4 days post-infection (dpi). Nucleotide sequences of the rescued virus genomes were verified by next-generation sequencing.

### 3.4. Assessment of Mutant Virus E2ΔCCDC115v Replication In Vitro

**Figure 3:**
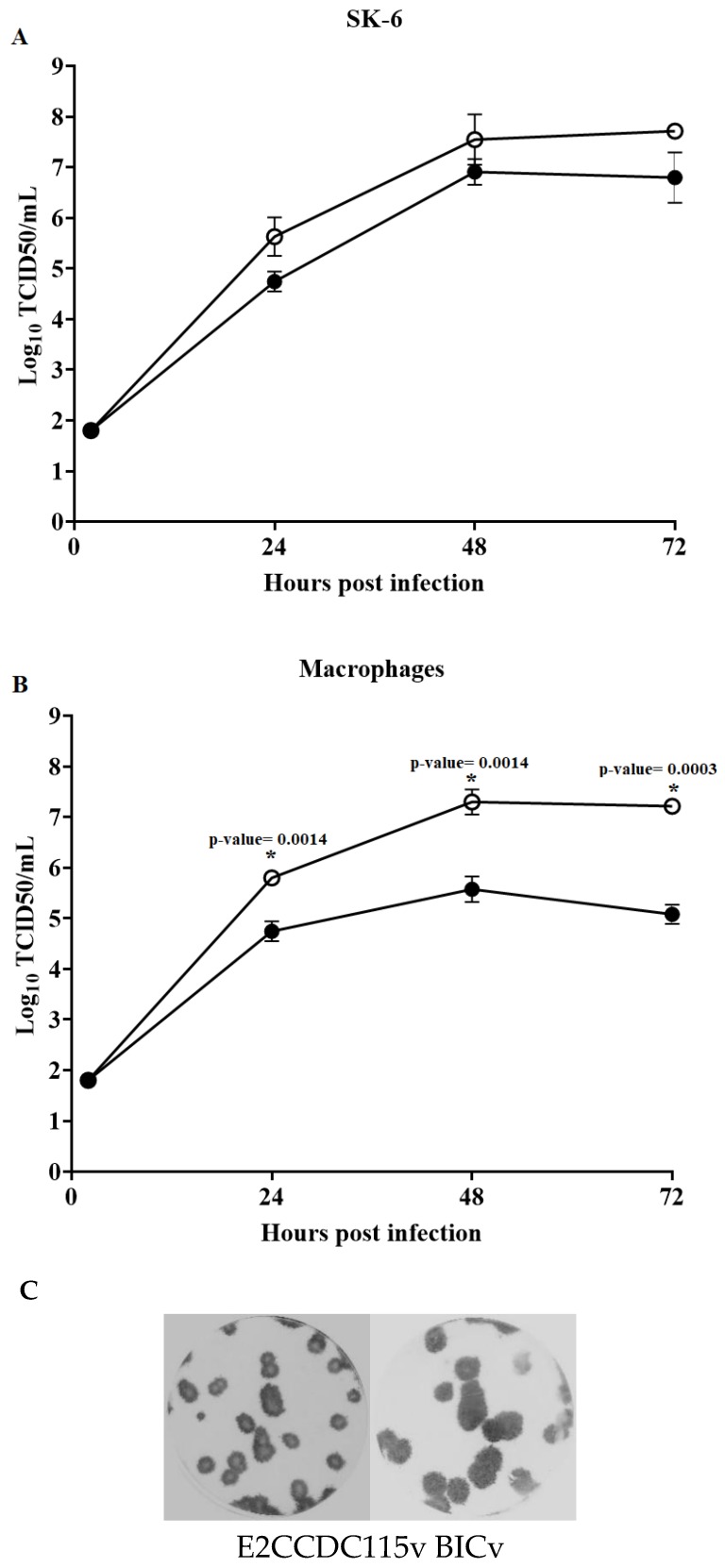
In Vitro growth characteristics of CSFV E2ΔCCDC115v. Multistep growth curve of E2ΔCCDC115v and BICv error bars represent the average and standard deviation for the titers of hree independent experiments on (**A**) SK6 cells and (**B**) swine macrophage cell cultures. Cell cultures were infected (MOI of 0.01) with CSFV BICv (empty symbols) or E2ΔCCDC115v (filled symbols). At indicated times post-infection, samples were collected and titrated for virus yield. Data are means and the bars represent the standard deviation of three independent experiments. Significant differences (*) in viral yields in SK-6 cells and macrophages between both viruses at specific times points were determined using the Holm–Sidak method (α = 0.05), without assuming a consistent standard deviation. All calculations were conducted on the software GraphPad Prism version 8. (**C**) Plaque formation of CSFV E2ΔCCDC115v and BICv on SK6 cell cultures. Cell cultures were infected with either virus, overlaid with 0.5% agarose, incubated at 37 °C for 4 days, fixed with 50% (*v*/*v*) ethanol/acetone, and stained by immunohistochemistry as described in Materials and Methods.

Growth characteristics of mutant virus E2ΔCCDC115v were assessed in both SK6 and primary swine macrophage cultures and were compared with that of parental BICv using a multistep growth curve. SK6 and primary swine macrophage cultures were infected at a multiplicity of infection (MOI) of 0.01 TCID_50_ per cell, virus was adsorbed for 1 h (time zero), and samples were collected daily until 72 hpi. As earlier reported, BICv replicates with similar efficiency both in SK6 cells and swine macrophages. E2ΔCCDC115v exhibited a slight reduction in virus replication in SK6 cells when compared with parental BICv and a significant decrease in virus yields in swine macrophages, with a reduction of approximately 10- to 100-fold when compared to that of BICv-infected macrophages at various sample times (Figure 3A,B).

Accordingly, with its decreased replication, plaque size produced by E2ΔCCDC115v in SK6 cell cultures was reduced by approximately 30–50% relative to that of BICv plaque size (Figure 3C). E2 residue substitutions shown to disrupt the E2–CCDC115 interaction in yeast two-hybrid clearly affected the ability of CSFV to replicate in primary swine macrophages cultures.

In addition, the ability of the mutated E2 protein to interact with CCDC115 was evaluated in swine macrophages infected with E2ΔCCDC115v. A statistically significant (*p*-value < 0.0008) reduction in the number of cells showing the E2–CCDC115 interaction was observed in the E2ΔCCDC115v-infected cells when compared with that observed in macrophages infected with the parental BICv (Figure 1B). 

### 3.5. Assessment of E2ΔCCDC115v in CSFV Virulence in Swine

**Figure 4:**
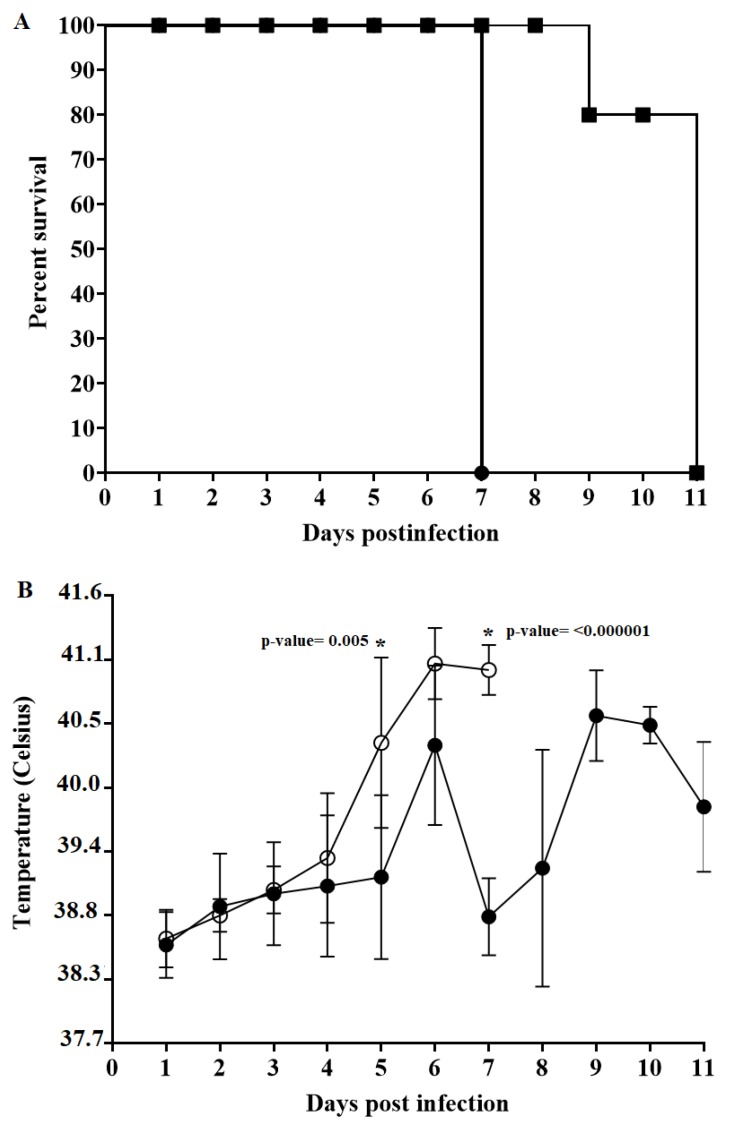
Evolution of mortality (**A**) and body temperature (**B**) in animals (five animals/group) IN infected with 10^5^ TCID_50_ of either E2ΔCCDC115v (filled symbols) or parental BICv (open symbols). Significant differences (*) in rectal temperatures between both groups of pigs infected with each virus at specific times post-infection were determined using the Holm–Sidak method (α = 0.05) without assuming a consistent standard deviation. All calculations were conducted on the software GraphPad Prism version 8.

To assess the potential effect of the disruption of the E2–CCDC115 interaction on CSFV virulence in vivo, naïve swine were inoculated intranasally (IN) with 10^5^ TCID_50_ of mutant E2ΔCCDC115v or parental BICv. Animals were then monitored daily for the appearance of clinical signs associated with the disease throughout a 21 day observational period. Animals infected with BICv displayed a well-characterized CSF disease, starting with a rise in body temperature by day 5 post-infection (pi) followed by the rapid appearance of classic clinical signs associated with CSF (Table 1 and Figure 4). Due to the severity of the disease, all BICv-infected animals were euthanized by day 7 pi. In contrast, animals infected with mutant E2ΔCCDC115v showed a delayed presentation of clinical disease. All animals presented a rise in body temperature by day 7–9 pi, with disease-associated symptoms progressing in severity in the following days making it necessary to euthanize one on day 9 pi and the others on day 11 (Table 1 and Figure 4). Survival curves were significantly different (*p*-value < 0.0001) between each group of pigs, as calculated using the Log-rank (Mantel–Cox) test (analysis was conducted on GraphPad Prism software version 8.2.1).

**Figure 5:**
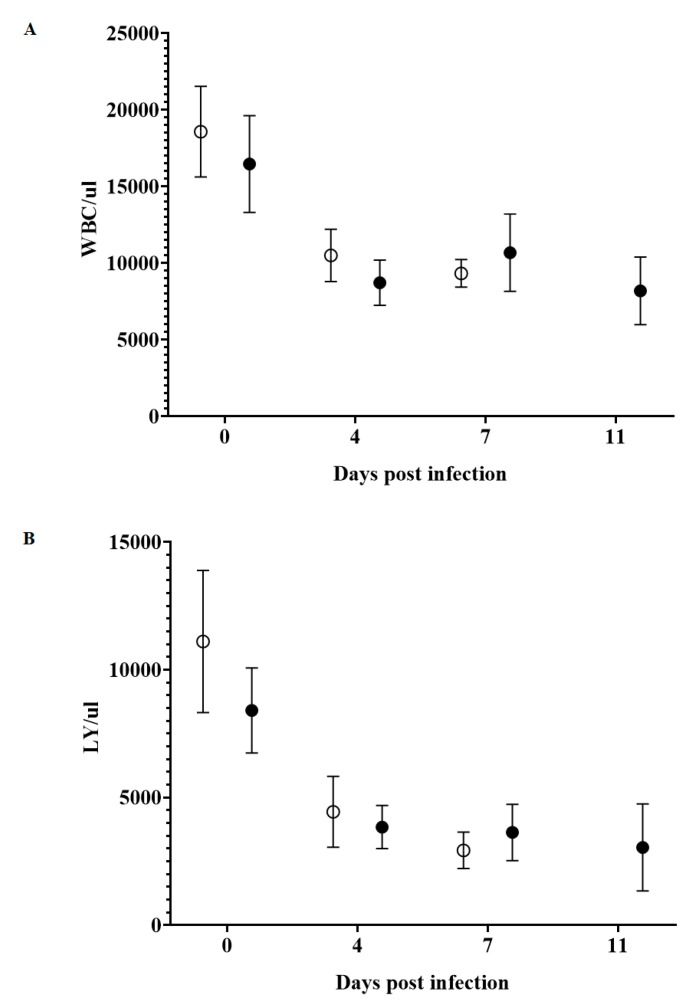

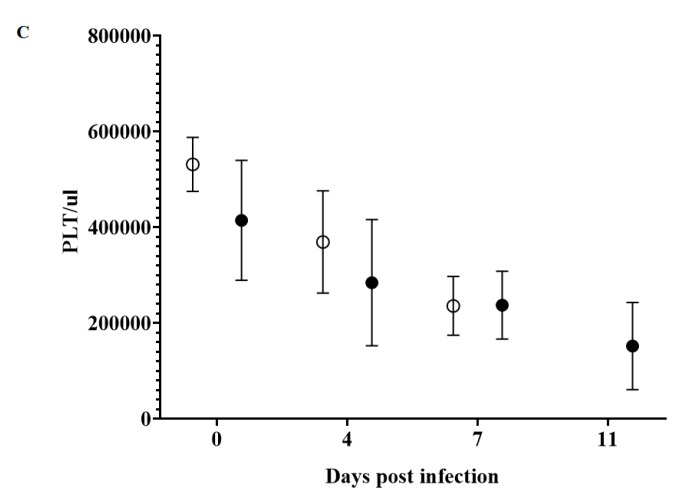
Concentration of circulating white blood cells (**A**), lymphocytes (**B**) and platelets (**C**) in animals IN infected with 10^5^ TCID_50_ of either E2ΔCCDC115v (filled symbols) or parental BICv (open symbols). Results represent the average and the standard deviation values of all animals in the group at the given time point. No significant differences were found between both groups of pigs at any time point using the statistical methodology described in Figure 3 and Figure 4.

The concentrations of circulating white blood cells (WBCs), lymphocytes and platelets were analyzed, as they are known indicators of CSF severity (Figure 5). In animals infected with BICv, circulating WBC counts significantly decreased by 4 dpi, and remained low until death by 7 dpi. Animals infected with E2ΔCCDC115v also showed a similar drop in circulating WBC values by day 4 pi and WBC values remained low until animals were euthanized. Similarly, circulating blood lymphocyte and platelet values in BICv-infected animals also dropped by day 4 and continued to decrease until animals were euthanized at 7 dpi. A clear decrease was also observed in blood lymphocytes and circulating platelet counts by day 4 and followed by lower counts at 9 dpi and 11 dpi (time when all animals were euthanized) in E2ΔCCDC115v-infected animals. As a summary, infection with E2ΔCCDC115v produced a decrease in hematological values statistically undifferentiated from that observed in the BICv-infected animals and values stayed low until the animal was euthanized. The decrease in blood cell counts was closely associated with the increased severity of clinical signs associated with the disease.

**Figure 6:**
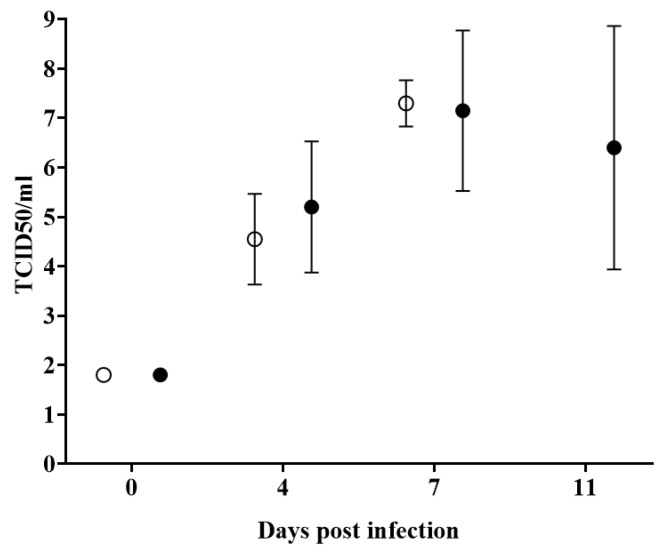
Virus titers in blood samples obtained from animals IN infected with 10^5^ TCID_50_ of either E2ΔCCDC115v (filled symbols), or parental BICv (open symbols). Values are expressed as log10 TCID_50_/_mL_ and error bars represent the average and the standard deviation of titers of all animals in the group at the given time point. Sensitivity of virus detection: ≥ 10^1.8^ TCID_50_/_mL_. No significant differences were found between both groups of pigs at any time point using the statistical methodology described in Figure 3 and Figure 4.

Virus replication in the infected animals of both groups, detected as viremia, closely followed the presentation of clinical signs. In BICv-inoculated animals, titers were clearly detectable (ranging between 10^4^ and 10^6^ TCID_50_/_mL_) by day 4 pi, increasing to higher values by day 7, when all the animals were euthanized. Virus titers in blood of animals inoculated with E2ΔCCDC115v were similar to those in BICv-inoculated animals by day 4 pi, increasing by day 7 pi, and remaining at similar levels until animals were euthanized. Interestingly, viremia values in the E2ΔCCDC115v-infected animals were heterogenous at each time point when compared to values obtained from animals infected with parental BICv (Figure 6).

In summary, animals infected with E2ΔCCDC115v presented with a significantly delayed progression of CSF disease, although their hematological values and levels of virus replication did not significantly differ in magnitude from those observed in animals infected with the parental virulent BICv strain.

## 4. Discussion

Interaction between virus and host proteins is a critical step in virus replication and, in some cases, disease production. Several reports detail the mechanisms used by CSFV to facilitate its replication involving specific host–virus protein–protein interactions. Through mechanisms mediated by interaction between virus and host proteins, the virus could modulate the host cell response, permitting the virus to manipulate host metabolic pathways to facilitate its replication. In that regard, we have already reported that structural CSFV Core protein interacts with host SUMO1, IQGAP1, UBC9 and OS9 [11,12,13,14]; the interaction of nonstructural protein p7, a viroporin, with CAMLG [28]; recently, we characterized the interaction of major structural glycoprotein E2 with host proteins PPP1CB and DCNT6 [20,21]. Interestingly, some of these host–virus protein–protein interactions also play a role determining virus virulence in swine [11,12,13,21]. Understanding the involvement of host factors in CSFV functions is important to further understand the molecular mechanisms associated with viral infection of the natural host. Here, we report the characterization of the interaction between CSFV E2 protein and CCDC115, a gene involved in the process of protein glycosylation in eukaryotic cells.

CCDC115 is a coiled-coil domain-containing protein, with a largely unknown cellular function. However, CCDC115 has been observed to localize to the endoplasmic reticulum-Golgi intermediate compartment and coat protein complex I vesicles in some human cells. The encoded protein shares some homology with the yeast V-ATPase assembly factor Vma22p, and the orthologous protein in mouse promotes cell proliferation and suppresses cell death. In humans, a missense mutation in the CCDC115 protein has been implicated to cause a disorder of Golgi homeostasis that causes abnormal protein glycosylation [23]. The Golgi function has been shown to be important for CSFV replication [31] and CSFV E2 glycosylation has been shown to be important for viral pathogenesis [8]. This suggests the possibility that E2 could be binding CCDC155 to aid in manipulating Golgi function or cellular glycosylation for its own advantage. Although the function of CCDC115 protein currently remains largely unknown, it is probable that E2 could be binding CCDC115 to manipulate downstream pathways to enhance virus replication and contribute to virus virulence.

The results reported here characterize, for the first time, cellular protein CCDC115 as an interaction partner for CSFV protein E2 in the infected cell. These results demonstrate that the E2–CCDC115 interaction is important for CSFV replication in cell cultures in vitro, particularly swine macrophages, the natural target cell during the infection in swine. Importantly, the E2–CCDC115 protein–protein interaction appears to play a role in virus virulence during CSFV infection in the natural host, the domestic swine. The same amino acid substitutions in E2 leading to the disruption of the E2–CCDC115 interaction in the yeast two-hybrid system when introduced into the virus clearly induce a decrease in virus virulence in swine. Animals infected with E2ΔCCDC115v present a significant delay in disease presentation and time of death. These results present new possibilities to explore CSFV pathogenesis and the virus requirements of host–virus interaction necessary to produce disease.

We previously described that the substitution of residue N260 partially affects E2 glycosylation, causing a shift in the elative molecular mobility of E2 in denatured Western blot [8]. A similar shift was observed in the E2 of SK6 cells and swine macrophages infected with E2ΔCCDC115v.

A more complete understanding of the host factors involved in virus replication in vivo and how these host factors are able to molecularly interact with E2 is an important step to gain a better understanding of the impact of potential mutations that occur between different emerging strains of CSFV (as have been described to occur recently), potentially helping to explain the differences observed in regard to viral virulence and pathogenesis.

## Figures and Tables

**Table 1:** Swine survival and fever response in animals infected with mutant E2ΔCCDC115v compared with those infected with parental BICv.

			Fever		
Treatment ^(1)^	No. of Survivors/Total	Mean Time to Death (Days ± SD)	No. of Days to Onset (Days ± SD	Duration; No. of Days (Days ± SD)	Maximum Daily Temp (°C ± SD)
BICv	0/5	7 (0)	5.2 (0.84)	1.8 (0.84)	40.01 (0.17)
CSFV E2ΔCCDC115v	0/5	(0.89)	8.6 (0.89)	1.6 (0.89)	40.62 (0.18)

^(1)^ All animals were intranasally (IN) inoculated with 10^5^ TCID_50_ of the indicated virus.
